# Ferroelectricity in Ultrathin HfO_2_-Based Films by Nanosecond Laser Annealing

**DOI:** 10.1021/acsami.4c10002

**Published:** 2024-10-03

**Authors:** Robin Athle, Megan O Hill, Austin Irish, Huaiyu Chen, Rainer Timm, Elias Kristensson, Jesper Wallentin, Mattias Borg

**Affiliations:** † Electrical and Information Technology, Lund University, Box 118, Lund 22 100, Sweden; ‡ NanoLund, Lund University, Box 118, Lund 22 100, Sweden; § Division of Synchrotron Radiation Research, Lund University, Box 118, Lund 22 100, Sweden; ∥ MAX IV Laboratory, Lund University, Box 118, Lund 22 100, Sweden; ⊥ Division of Combustion Physics, Lund University, Box 118, Lund 22 100, Sweden; # Lund Laser Center, Lund University, Box 118, Lund 22 100, Sweden

**Keywords:** hafnium oxide, thin films, ferroelectric, FeRAM, BEOL compatibility

## Abstract

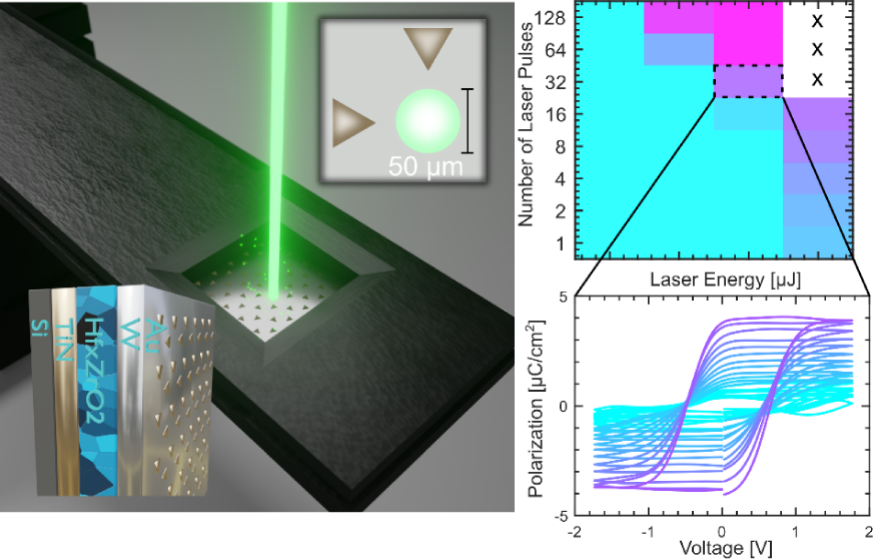

Nonvolatile memory devices based on ferroelectric Hf_*x*_Zr_1–*x*_O_2_ (HZO) show great promise for back-end integrable storage and for neuromorphic accelerators, but their adoption is held back by the inability to scale down the HZO thickness without violating the strict thermal restrictions of the Si CMOS back end of line. In this work, we overcome this challenge and demonstrate the use of nanosecond pulsed laser annealing (NLA) to locally crystallize areas of an ultrathin (3.6 nm) HZO film into the ferroelectric orthorhombic phase. Meanwhile, the heat induced by the pulsed laser is confined to the layers above the Si, allowing for back-end compatible integration. We use a combination of electrical characterization, nanofocused scanning X-ray diffraction (nano-XRD), and synchrotron X-ray photoelectron spectroscopy (SXPS) to gain a comprehensive view of the change in material and interface properties by systematically varying both laser energy and the number of laser pulses on the same sample. We find that NLA can provide remanent polarization up to 2*P*_r_= 11.6 μC/cm^2^ in 3.6 nm HZO, albeit with a significant wake-up effect. The improved TiN/HZO interface observed by XPS explains why device endurance goes beyond 10^7^ cycles, whereas an identical film processed by rapid thermal processing (RTP) breaks already after 10^6^ cycles. All in all, NLA provides a promising approach to scale down the ferroelectric oxide thickness for emerging HZO ferroelectric devices, which is key for their integration in scaled process nodes.

## Introduction

In recent years, ferroelectric HfO_2_ has emerged as a promising material for nonvolatile memory applications.^1^ Its CMOS compatibility and excellent scalability provide a solution to the challenges that have hindered the adoption of perovskite-based ferroelectrics in CMOS applications. The alloy between HfO_2_ and ZrO_2_, Hf_*x*_Zr_1–*x*_O_2_ (HZO), has quickly become the most promising choice for back end of line (BEOL) integration due to its wide doping window and relatively low crystallization temperature.^1−4^ The discovery of ferroelectric HfO_2_ has made it possible to embed ferroelectric random access memory (FeRAM), ferroelectric field-effect transistors (FeFETs), and ferroelectric tunnel junctions (FTJs) onto CMOS chips.^5−7^ FTJs, in particular, are highly promising for ultradense storage and in-memory computing accelerators but struggle with a low current density that hinders device scaling and prevents fast readouts.^5,8^ This is a challenge that can be addressed by reducing the thickness of the ferroelectric barrier as the tunneling current is exponentially dependent on this thickness. Similarly, FeRAM and FeFETs have the potential to become energy-efficient replacements for dynamic random access memory (DRAM) due to being nonvolatile memories with ultralow switch energy.^5^

However, the high coercive field of fluorite-based ferroelectrics in comparison with conventional perovskite ferroelectrics comes at the cost of higher voltages needed to switch the polarization state. To be compatible with the operating voltages of scaled CMOS nodes, it is therefore crucial to scale down the ferroelectric thickness and thereby the switching voltages. In this work, we demonstrate scaling of HZO films down to 3.6 nm, with switching voltages as low as 0.5 V, while maintaining a BEOL-compatible thermal process.

While ferroelectricity is achievable in ultrathin HfO_2_, it is extremely challenging to integrate into the CMOS BEOL process flow.^16^ It is now accepted that in HfO_2_, ferroelectric properties arise from the orthorhombic Pca2_1_ crystal phase (o-phase); however, crystalline HfO_2_ can exist in many other phases such as monoclinic (m), cubic (c), and tetragonal (t). Which phase is the most stable depends on a range of factors including film thickness, film stress, grain size, and the thermal process.^17^ Typically, the o-phase is formed by first crystallizing into the t-phase, followed by a t→o phase.^18,19^ At a film thickness below ∼5 nm, the required temperature to initiate crystallization into the t- and o-phases increases rapidly, primarily due to the increasing surface and interface crystallization energies in ultrathin films, a trend well-documented in the literature.^3,20−24^ Elevated temperatures pose a threat to the integrity of the digital circuits fabricated in the front end of line (FEOL), which is a major hurdle for BEOL integration of HfO_2_-based ferroelectrics.^25,26^ For the 130 nm CMOS process node, a substrate temperature below 500 °C is required to maintain BEOL compatibility.^11,27,28^ This may not preclude the use of HZO films 10 nm thick or above, for which the required annealing temperature is in the range of 300–450 °C.^3,4,29^ However, films below 5 nm typically require much higher temperatures to crystallize. Examples of FTJs fabricated with 4.5 nm HZO required annealing temperatures of 525 and 600 °C, respectively.^30,31^ Cheema et al.^32^ and Jo et al.^20^ each achieved ferroelectric HZO as thin as 1 nm, but annealing temperatures up to 1000 °C were used, impeding BEOL integration.

Nanosecond laser annealing (NLA) has emerged as a promising alternative to overcome the challenges associated with detrimental annealing temperatures. Table 1 provides a summary of previous studies published on NLA of HfO_2_-based thin films, demonstrating the viability of the approach.^9−15^ In NLA, the annealing takes place in the nanosecond regime, confining the heat to the top few hundred nanometers in the device stack, limiting the thermal impact on underlying devices. Additionally, with heating and cooling rates at nanosecond time scales, diffusive and dissociative processes are greatly reduced in comparison with conventional RTP potentially limiting the degradation of the metal-oxide interface often seen in RTP processes.^33−36^

**Table 1:** Summary of the Literature on Laser-Induced Crystallization of Ferroelectric Hafnia Compounds

Reference	Stack	Thickness FE (nm)	*P*_r_ (μC/cm^2^)	Switching voltage (V)^a^	Laser type and wavelength	Pulse length
Ali et al.^9^	TiN/HSO/TiN	10	12	1	XeCl 308 nm	160 ns
Song et al.^10^	TiN/HZO/TiN	10	10	1	KrF 248 nm	20 ns
Grenouillet et al.^11^	TiN/HSO/TiN	10	9.5	1	XeCl 308 nm	160 ns
Chen et al.^12^	TiN/HZO/TiN	8	10	0.8	KrF excimer 248 nm	20 ns
Volodina et al.^13^	TiN/HZO/W	10	25	1	Nd:YAG 1064 nm	1 ms/16 ns
Tabata et al.^14^	HfO_2_/TaN	10	2.5	1	UV laser 300–400 nm	26 μs
Frechilla et al.^15^	TiN/HZO/TiN	10	6	1	Nd:YAG 1064 nm	800 ps
**This work**	W/HZO/TiN	**3.6**	5.8	**0.5**	Nd:YAG 532 nm	6 ns

a Based on an *E*_c_ of 1 MV/cm.

However, the applicability of NLA to HfO_2_ less than ∼8 nm has not been considered previously. In this work, we investigate the use of NLA to crystallize ultrathin HfO_2_ (3.6 nm), whose thickness can allow for higher current levels in FTJs and lower operation voltages required for CMOS scaling. Using NLA, we attempt to crystallize the ferroelectric o-phase in such films while maintaining a BEOL-compatible temperature within the larger device stack. Additionally, we directly characterize the laser-annealed regions using both nanofocused X-ray diffraction (nano-XRD) and X-ray photoelectron spectroscopy (XPS) providing crucial insights into the structural and chemical effects of the NLA process at the nanoscale level.

## Results

### BEOL-Compatible Ultrathin Hf_*x*_Zr_1–*x*_O_2_ by NLA

**Figure 1:**
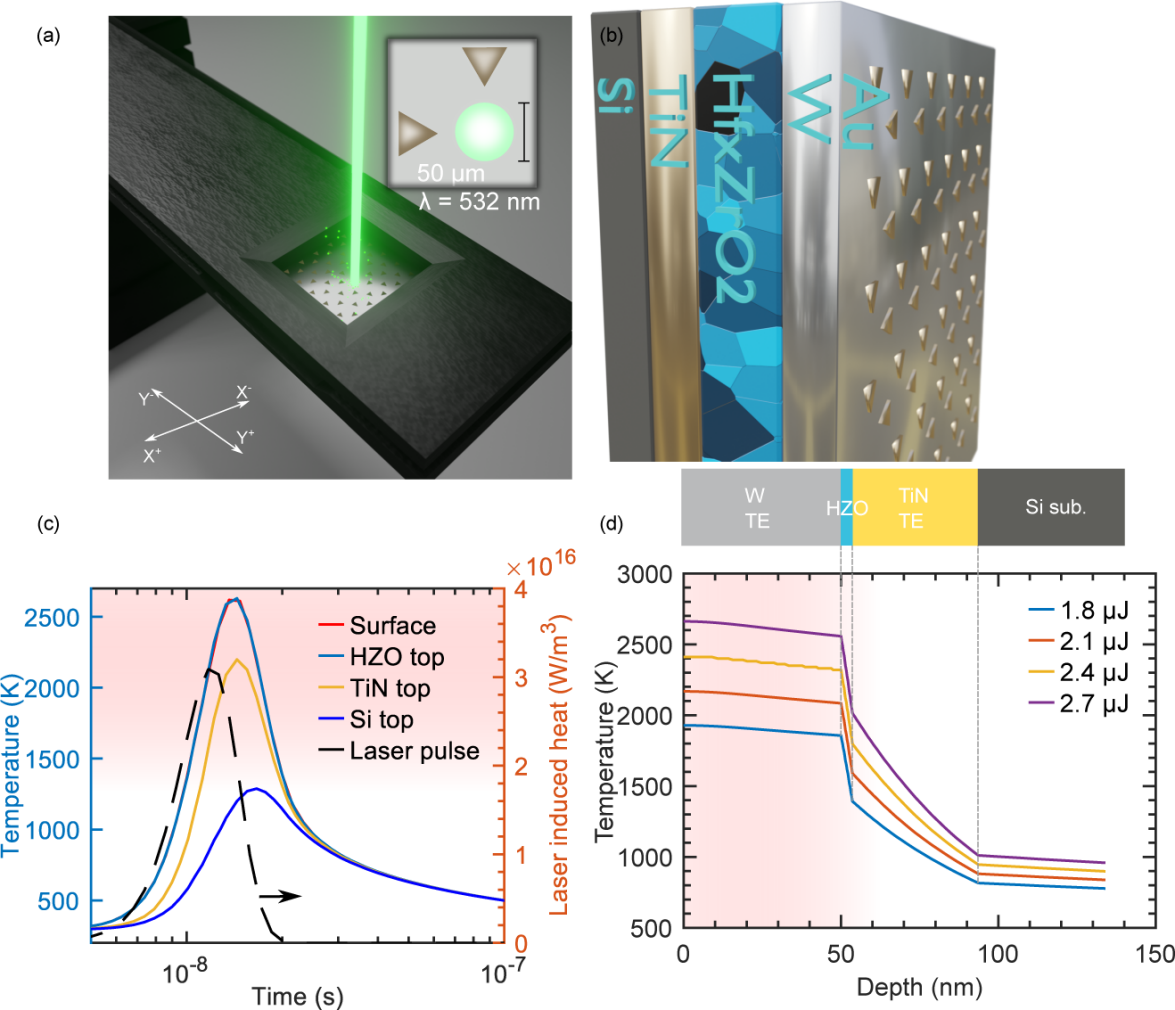
Nanosecond laser annealing setup and modeling. (a) Schematic of the NLA setup with the green YAG laser with λ = 532 nm and a spot size of 50 μm which is used to locally anneal the sample surface. (b) Device stack schematic of the capacitor structures; the golden triangles are used as reference coordinate markers for alignment of the laser spot during the annealing. (c) 1D thermal simulation of the temperature evolution with time for the top part of different layers in the device stack, and (d) shows a cross-section plot of the maximum temperature along the depth of the stack for pulse energies from 1.8 to 2.7 μJ.

The NLA setup in this work is shown in Figure 1a and provides highly confined laser annealing due to the small spot size with a diameter of around 50 μm. The TiN/HZO/W metal–insulator–metal (MIM) capacitor structure evaluated is depicted in Figure 1b, with Au markers for aligning the laser spot during the NLA treatment. Details on the fabrication steps are described in the Methods section. Figure 1c shows the result of finite element (FEM) simulations of the temperature propagation with time upon exposure to the laser pulse at the top interface of each device layer (details of the simulation are provided in the Supporting Information). Additionally, in Figure 1d, the simulated cross-sectional temperature profile along the depth of the stack for varying NLA energies of 1.8–2.7 μJ is shown. FEM simulations show that the nanosecond laser pulse should be completely absorbed in the top W layer (Figure S1a), and, apart from reflections, the ultrashort pulse duration results in near-ideal energy deposition in this layer, without time for heat diffusion. Thus, temperatures above 2000 K can be achieved in the HZO layer, while keeping the Si substrate temperature below 800 K, demonstrating the desired BEOL compatibility. By adding a 100 nm SiO_2_ spacer, the Si substrate temperature can be reduced even more, to 430 K, as demonstrated in Figure S1, along with details on the temperature simulations.

### Electrical Characterization of NLA-Treated Capacitors

**Figure 2:**
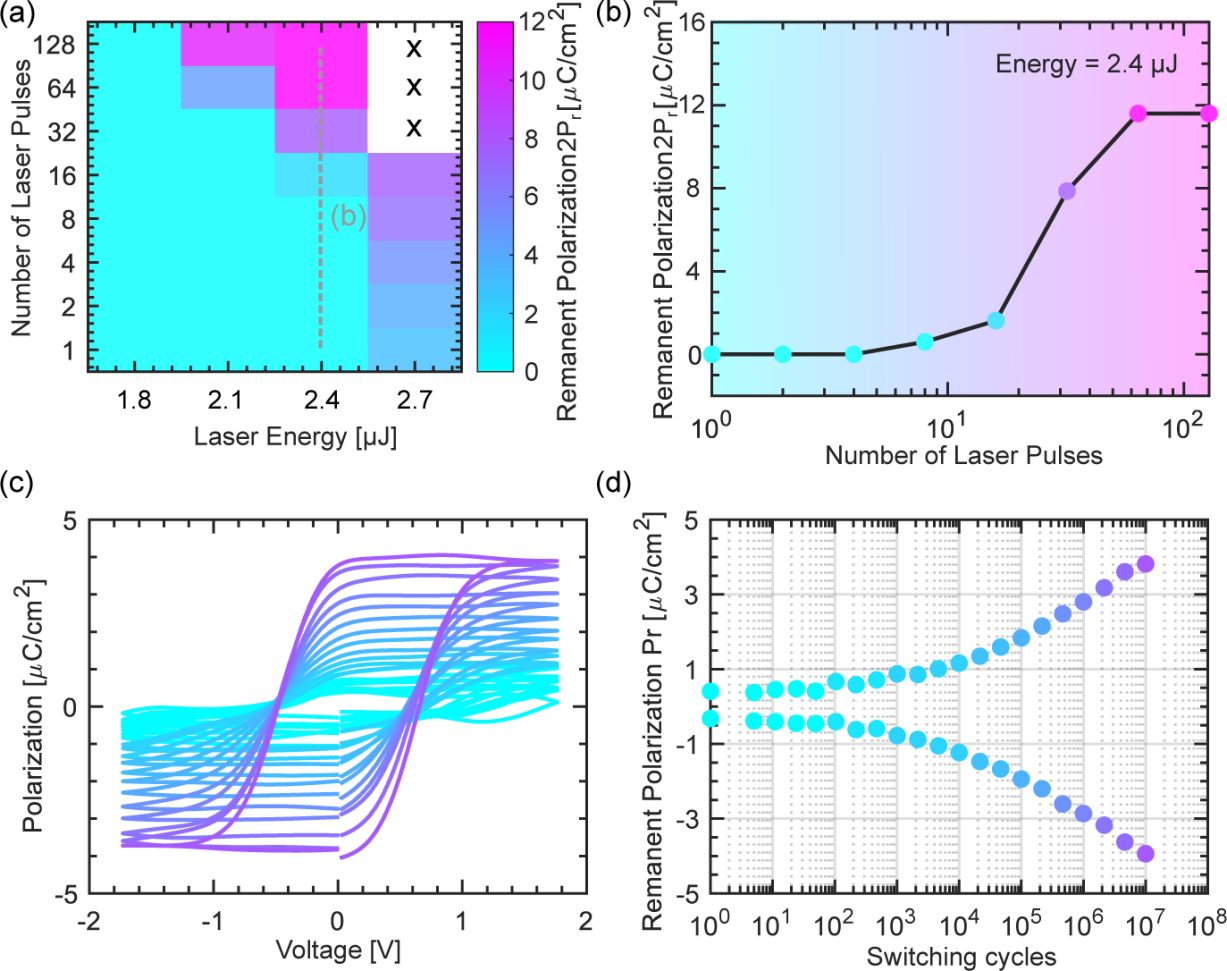
Electrical characterization of the ferroelectric capacitors. (a) Summary table of the measured remanent polarization for different laser intensities and numbers of pulses during the NLA treatment. The dashed gray line indicates the data extracted in (b). (b) The measured remanent polarization as a function of the increasing number of laser pulses for a laser energy of 2.4 μJ. (c),(d) The evolution of the polarization-electric field hysteresis curve with cycling and the extracted remanent polarization.

The engineered coordinate system on our chips allows us to precisely track the location of each NLA exposure condition. This allows for capacitor fabrication and correlation between process conditions, electrical properties, and nano-XRD and XPS data. The electrical characterization of the NLA devices is presented in Figure 2. Figure 2a shows a map correlating the measured remanent polarization 2*P*_r_ for laser energy ranging from 1.8 to 2.7 μJ and the number of laser pulses from 1 to 128. The 2*P*_r_ increases toward the upper right corner; however, at an energy of 2.7 μJ, devices exposed to 32 laser pulses or more become short-circuited (marked with x in the figure). This confirms that the ultrafast annealing of only 6 ns is sufficient to induce the ferroelectric phase in the HZO, although 2*P*_r_ = 3 μC/cm^2^ is lower than what is routinely achieved in thicker films using traditional RTP (∼20 μC/cm^2^).^37,38^ However, it is in line with previous reports on NLA^10−12,14,15^ and FLA.^36,39^ Lower 2*P*_r_ values are likely an effect of the ultrashort annealing duration, which is sufficient to initiate crystallization but does not allow time for grain growth. This means that while the ferroelectric o-phase is likely beginning to crystallize, a relatively large fraction of the film may remain amorphous.

Repeatedly pulsing the laser beam leads to higher 2*P*_r_ values, as evident from the map in Figure 2a, and also shown in Figure 2b for a laser energy of 2.4 μJ, possibly allowing the grains to grow with subsequent laser pulses. For an energy of 2.4 μJ, a clear improvement in 2*P*_r_ is observed with increasing laser pulses above eight, saturating at a maximum 2*P*_r_ = 11.6 μC/cm^2^ after 64 laser pulses. Crystallization of an identical RTP sample requires a temperature of 700 °C for 30 s to yield a somewhat larger 2*P*_r_ = 20 μC/cm^2^ (Figure S2) with the drawback that such a process would not fit within the thermal restrictions of a BEOL-compatible process.

Finally, in Figure 2c,d, the evolution of the polarization-electric field curve and remanent polarization with cycling is presented, respectively. The P–E curve of the device with the largest 2*P*_r_ is shown in Figure S3. A persistent wake-up effect is present in the NLA-treated films, which exhibit increasing remanent polarization past the measured 10^7^ cycles. This is consistent with a field-induced t→o phase transition.^40^ It is worth noting that NLA devices achieve superior endurance with at least 10^7^ cycles, in contrast to the RTP reference sample on which the capacitors break down after about 10^6^ cycles (Figure S2b). The improved endurance obtained by the NLA process is an indication of improved film and/or interfacial properties, as further discussed in the XPS section below.

### Local Mapping of Ferroelectricity in Ultrathin Hf_*x*_Zr_1–*x*_O_2_

**Figure 3:**
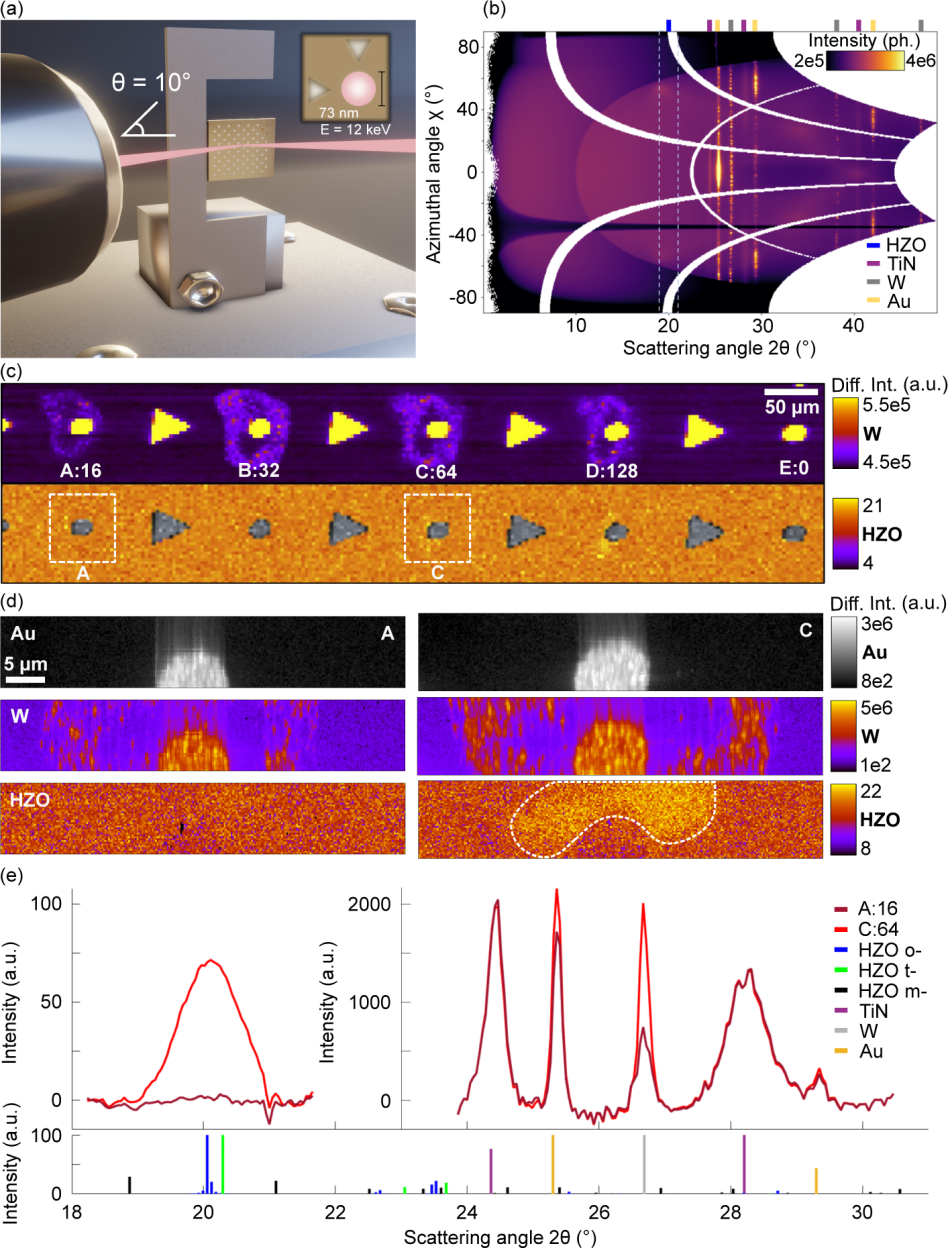
Nanoprobe scanning X-ray diffraction (nano-XRD) of ferroelectric Hf_*x*_Zr_1–*x*_O_2_. (a) A schematic of the measurement setup where hard synchrotron X-rays with an energy of 12 keV are focused down to a spot size of 73 nm × 73 nm, which are scanned along the sample (not a surface measurement) to allow 2D mapping of the crystallography. (b) Integrated diffraction data for one map, shown as a function of the azimuthal angle χ and scattering angle 2θ. Expected diffraction angles are marked for HZO, TiN, W, and Au. (c) Coarse mapping XRD of W(110) diffraction intensity (top) labeling contacts with a letter and a corresponding number of laser pulses (i.e., A:16, exposed to 16 laser pulses) and (bottom) HZO scattering (2θ between 18.5° and 21.5°). Au signal from top contacts is overlaid in gray for reference. A weak HZO signal is observed near contacts C and D; ROIs around contacts A and C are marked. (d) High-resolution XRD scans of devices A and B, left and right, respectively. Diffraction intensity is shown for Au (top), W (middle), and HZO (bottom). The dotted lines (right, bottom) correspond to the approximate ROI for the integration of scattering plot in (e). (e) Integrated line scan from diffraction patterns for C:64 using pixels with HZO signal above 70% of the maximum signal (approximately corresponding to the ROI marked by white dashes in (d)). An equivalent area is used for A:16. Bar graph (bottom) shows the simulated powder diffractograms of cubic TiN/W/Au, monoclinic HfO_2_, orthorhombic HZO, and tetragonal HfO_2_.

Characterizing the amorphous to crystalline transition in ultrathin ferroelectric HZO is very challenging, particularly since the NLA process leads to localized crystallization (<50 μm crystalline region). X-ray diffraction in grazing incidence geometry (GIXRD) is a well-established technique to characterize the crystal phases in ferroelectric polycrystalline films;^41,42^ however, GIXRD does not provide the spatial resolution required to probe crystallization within the <50 μm laser spot. Transmission electron microscopy, on the other hand, has been used to identify crystal phase variations in ultrathin HZO, but this requires potentially destructive ion-beam milling procedures and can only probe a very local region (<1 μm).^20^ Lab-based microdiffraction can be used in the diffraction geometry (not in the GIXRD geometry) to achieve higher spatial resolution. However, in these geometries, lab-based microprobes do not produce sufficient flux to characterize films that are below 5 nm thick. Here, we instead utilize synchrotron-based nanofocused scanning X-ray diffraction (nano-XRD) which provides 2D spatial diffraction maps. Figure 3a shows a schematic of the nano-XRD setup with the high-flux synchrotron beam incoming at an incidence angle of 10°, an energy of 12 keV, and an X-ray probe size of 73 nm × 73 nm. The incident angle of 10° was chosen to optimize scattering from the highly textured (111) HZO orthorhombic phase (2θ ∼ 20° at 12 keV). Scattering at 10° corresponds to a beam footprint of approximately 73 nm × 415 nm. Figure 3b shows the wide-angle scattering collected during a measurement. Coarse spatial maps were taken across five devices, A–E. Devices A–D were exposed to NLA pulses with an energy of 2.1 μJ with an increasing number of laser pulses (16, 32, 64, and 128 for A–D, respectively), while E was kept unprocessed as a pristine reference. Figure 3c presents a coarse map of the W(110) diffraction (top), in which each pixel corresponds to the summed W(110) scattering intensity from a single diffraction pattern collected at that probe position. In the W(110) image, three features are present: Au/W alignment markers (triangular), Au/W device contacts, and a weaker outer ring of W diffraction. This outer W ring is approximately the diameter of the laser spot and is likely an effect of Au diffusion during NLA (Supporting Information). Figure 3c (bottom) shows the scattering intensity between 18.5° and 21.5°, isolating the signal from HZO. This has been overlaid with Au markers (in gray) for clarity. Small regions of slightly increased scattering intensity can be seen near devices C and D.

In Figure 3c (bottom), two regions of interest (ROIs) are defined around devices A (2*P*_r_ = 0 μC/cm^2^) and C (2*P*_r_ = 4.6 μC/cm^2^), which are investigated at higher resolution. Figure 3d presents the high-resolution XRD maps for device A (left) and device C (right), showing diffraction intensity of Au(111), W(110), and HZO (18.5° < 2θ < 21.5°). The vertical smearing comes from the asymmetric footprint of the beam. There is distinct scattering intensity from the HZO reflection in C, in the form of an asymmetric ring around the Au/W device contact. Though we also expect the HZO to crystallize under the contact pad, the background signal from W and Au scattering overwhelms the weak HZO signal so the HZO peak cannot be resolved in this area. The spatial resolution provided by the nanoprobe allows for the differentiation of the XRD signal that comes from the laser annealing spot versus beneath the metal electrodes, allowing us to determine the effect of laser annealing on crystallization, ruling out effects of device cycling. For A, where only 16 laser pulses were used, no measurable intensity change is observed, indicating that the HZO remains amorphous in this area. Given the ultralow thickness of the HZO, individual diffraction patterns are very weak; therefore, to look at the HZO diffraction, we sum over multiple probe positions. For device C, all pixels with HZO scattering above 70% of the maximum HZO scattering are averaged. This roughly corresponds to the ROI marked by white dashes in Figure 3d (left, bottom). An equivalent area is summed for device A. The scattering from these regions is plotted as integrated line scans in Figure 3e. Inlaid below the integrated diffraction signal in Figure 3e are the expected diffraction peaks for m- and t-HfO_2_, o-HZO, and cubic TiN, Au, and W. A strong HZO peak near 20° is observed for device C but not for device A. This peak overlaps with the expected peak positions for o-phase (111) HZO and t-phase (011) HfO_2_. While the t-phase and o-phase peak overlap is too large to precisely deconvolve the percentage of each, it can be said that regardless of fit parameters, the o-phase contribution is stronger than that of t-phase (see Figure S4). Thus, we conclude that the NLA process has locally crystallized HZO with a large share of the o-phase.

As further confirmation of the spatial selectivity of the NLA process, piezoresponse force spectroscopy (PFS) measurements were performed at two regions I and II in close proximity to the electrode of devices C and A (Figure S5). In region I, where HZO crystallization was observed by nano-XRD, we observe a typical ferroelectric response in amplitude and phase, with a phase shift of 180° and coercive voltage around 1 V, indicative of the ferroelectric properties of the HZO in this region. In contrast, in region II, no phase loop hysteresis is observed, and the amplitude response is basically DC bias independent, consistent with the indication from the nano-XRD data that the HZO film in this region is amorphous.

### HZO/TiN Interface Properties

**Figure 4:**
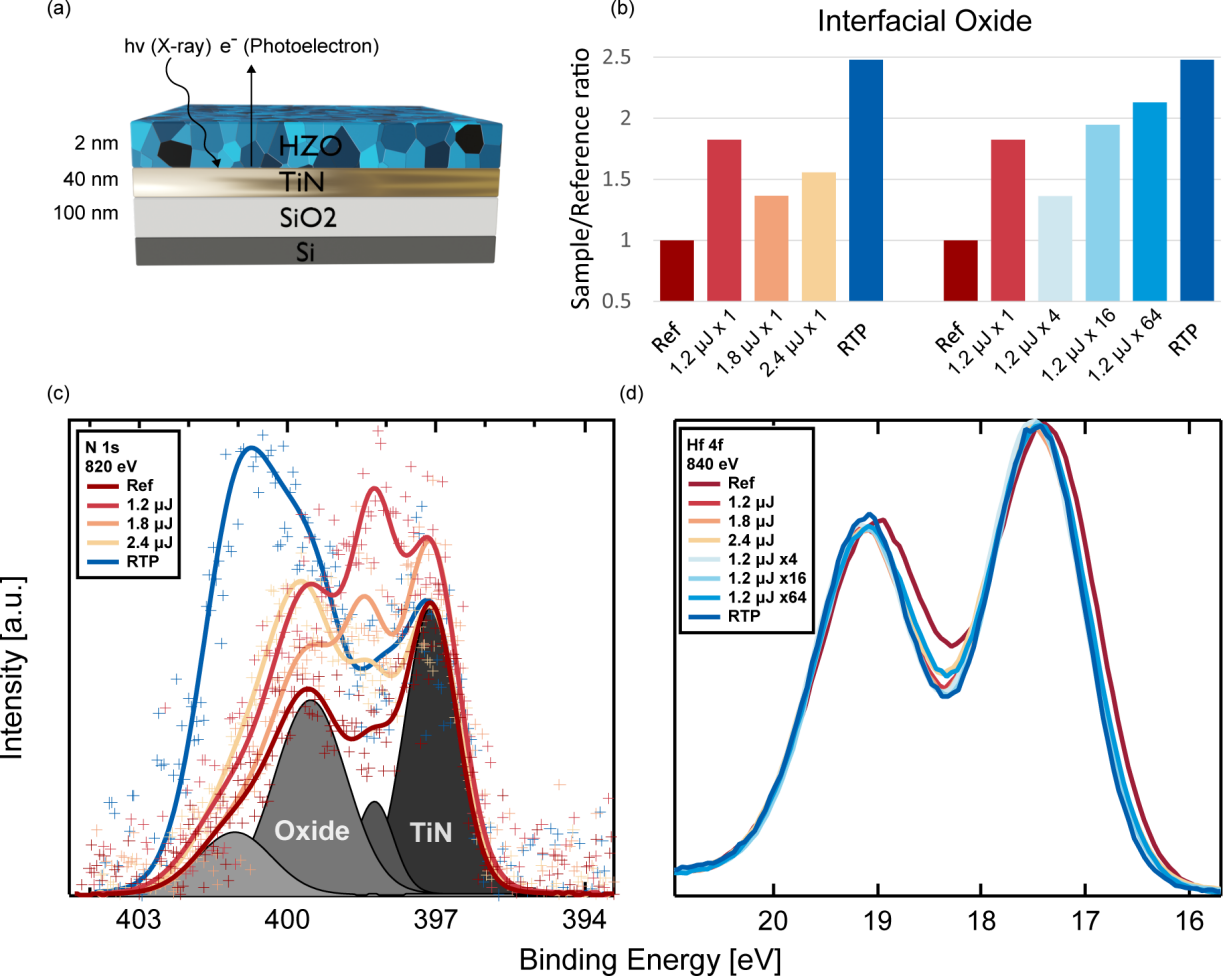
XPS characterization of Hf_*x*_Zr_1–*x*_O_2_/TiN interface chemistry. (a) Schematic of thin films and XPS probing depth. (b) N-oxide intensities extracted from N 1s fitting, normalized to an unannealed reference region. (c) N 1s core level spectra showing nitrogen species probed near the HZO/TiN interface with a photoelectron kinetic energy (KE) of 420 eV, resulting in an inelastic mean free path (IMFP) of 1.1 nm. (d) Hf 4f core level spectra probed with KE = 820 eV, IMFP = 1.7 nm.

A pronounced issue in HZO-based ferroelectric devices is the formation of parasitic dielectric layers at the electrode/ferroelectric interface which degrade performance.^35^ For TiN electrodes, the formation of an oxide or oxynitride as a result of oxygen scavenging at elevated temperatures is commonly reported.^35^ We expect NLA to strongly suppress such metal-oxide interface degradation, in contrast to conventional annealing approaches including RTP, since the time scale of NLA is orders of magnitude shorter than the diffusive processes involved in interfacial chemistry. To verify this, we employed XPS and evaluated the chemical characteristics of ultrathin HZO films and their interfaces with TiN in areas exposed to NLA. In these experiments, we limited the HZO film thickness to 2 nm (Figure 4a) to accommodate for the surface sensitivity of XPS and allow for the characterization of the buried TiN/HZO interface. Also, this thin HZO film was strained by depositing a W top electrode, which was removed by wet etching after the annealing, in the same way as the samples for electrical characterization (see the Methods section for details). For the XPS characterization, the micropositioning system at the FlexPES beamline with an approximately 30 μm narrow X-ray beam at the MAX IV synchrotron in Lund, Sweden, was combined with our engineered coordinate system to systematically probe interface properties of varying NLA treatments and compare them to those of RTP and unannealed, as-deposited reference HZO films. We used N 1s and Ti 2p core levels to characterize transformations of the buried interface. Hf 4f, O 1s, and Zr 3d core levels were monitored to understand the influence of annealing on the active HZO layer. While changes observed in Hf 4f indicated a similar thermal ordering/crystallization of the HZO for all annealed samples, N 1s spectra allowed us to specifically correlate degradation of the HZO/TiN interface with the long time scale (seconds) associated with typical RTP heating (see Figure 4b).

X-ray photoelectron spectra in the region of N 1s are shown in Figure 4c and contain contributions which we ascribe to TiN at a binding energy of 397.1 eV and two indeterminate nitrogen-containing oxide components at 398.2 and 399.5 eV, in accordance with the literature.^43,44^ Despite the short time scales, NLA crystallization inevitably increases the dissociation of TiN bonds at the interface and the possibility for reaction with nearby oxygen. This aligns with previous studies of oxygen scavenging by TiN during annealing.^45^ Though all samples, including the reference sample, had oxide located at the HZO/TiN interface, these oxide components are found to increase with both time and thermal energy during annealing. An additional component in the N 1s spectra was observed at 401 eV and was pronounced only in the RTP sample. We agree with previous studies which assign this to a high oxidation state form of nitrogen, considering that molecular nitrogen is typically located near 402 eV.^46^ The RTP sample furthermore stands out as showing much more oxide generally and less TiN than the NLA samples. This is consistent with our understanding of the annealing processes themselves. Unlike with NLA, since RTP maintains the entire sample at an elevated temperature outside of a vacuum for tens of seconds, there exists both the energy and time necessary for the interface to dissociate and for atoms to diffuse and react. Results from fitting and quantification of the various chemical species in the N 1s spectra are highlighted by the trend in Figure 3b and summarized more systematically in Table S1 and Figure S6. Besides the significant impact of RTP on the interface, these results also reveal the influence of the NLA parameters on interfacial oxide formation. Importantly, within NLA, there exists a parameter space that can be tuned to suppress the degradation of the interface. Whereas lower laser energy treatments with few laser pulses like 2.1 μJ x1, 2.4 μJ x1, and 1.2 μJ x4 only increased the oxide visible to XPS by 50% relative to the as-grown sample, higher energy treatments using 16 or more pulses at 1.2 μJ contributed to double or more of the interfacial oxide with RTP being the worst. Though the signal from the N 1s XPS data comes mostly from the topmost part of the TiN layer and highlights the TiN/HZO interface, results obtained from Hf 4f spectra as shown in Figure 4d can be attributed to the entire HZO film. From Figure 4d, we note a significant narrowing of the full width at half maximum (fwhm) of the Hf 4f spectra from 1.4 to 1.2 eV for all samples following annealing, as compared to the reference sample. This represents a compositional ordering throughout the HZO film due to heating at all time scales. As others have reported,^47^ we attribute this to crystallization since the transformation is irreversible and precludes interaction with ambient. It should be noted that the same laser energies and number of pulses can have different influences on the interfaces of the samples used for XPS and for nano-XRD and electrical measurements due to the different sample structures, especially the much thinner HZO in the XPS samples. Still, the trends observed by nano-SXRD and XPS confirm each other. Photoelectron spectroscopy makes it clear that NLA parameters can be selected to suppress the growth of an interfacial oxide layer, especially the thermal and diffusive processes leading to high oxidation state nitrogen compounds at the TiN/HZO interface, which are unavoidable with RTP.

## Conclusions

Our work demonstrates the use of NLA as a viable approach to crystallize ultrathin ferroelectric HZO films below 4 nm thickness while limiting the required thermal budget to enable the integration of ferroelectric devices in BEOL. By using a laser spot of 50 μm, spatial selectivity in the annealing can be achieved, which opens up possibilities for heterogeneous device integration on the same chip. The spatial selectivity of the NLA approach is verified by nano-XRD, which confirms the localized crystallization of the HZO. In addition, synchrotron-based XPS confirms the significant suppression of diffusive and dissociative processes at the TiN/HZO interface, which has significantly reduced Ti oxidation compared to a reference RTP sample. This is credited to the nanosecond time scale of NLA. Finally, by electrical characterization, the ferroelectric properties of the HZO are quantified, in which we find substantial values of 2*P*_r_ and cycling endurance beyond 10^7^ cycles, more than 10x higher than the RTP reference. The remaining challenges with NLA include a persistent wake-up effect and lower 2*P*_r_ values compared to RTP, which are attributed to small grain sizes. Even so, the successful demonstration of ferroelectric HZO in sub-4-nm films using NLA is a major step toward their integration in the BEOL of scaled process nodes, both for high-performing FeRAM, FeFET, and FTJ memory and neuromorphic devices.

## Methods

### Device Fabrication

A 40-nm-thick TiN bottom electrode (BE) was deposited on a 2 in. Si(100) by RF sputtering at a power of 150 W in an AJA Orion system. Subsequently, 40 cycles (∼3.6 nm) of Hf_*x*_Zr_1–*x*_O_2_ (20 cycles for XPS samples) were grown by alternating cycles (1:1) of TEMA(Zr) and TDMA(Hf) precursors by thermal atomic layer deposition (ALD) at 200 °C, with water as the oxidizing precursor. For all samples, a 50 nm W top electrode was deposited on top of the HZO films using DC magnetron sputtering at 100 W to induce tensile strain in the HZO and promote the formation of the ferroelectric phase. The 2″ wafer was then split into 1 × 1 cm large samples. On the NLA sample, a reference coordinate system was deposited using UV-lithography and a liftoff process of Ti/Au (5/200 nm). Post-metallization annealing (PMA) by NLA was carried out using an LCS-4 532 YAG laser with λ = 532 nm and a pulsing frequency of 1 Hz. The Gaussian beam was focused on optical lenses to a spot size of around 50 μm, and no scanning was used. Post PMA, a probing electrode of 200 nm Au was deposited by using UV-lithography and liftoff. Finally, the capacitors were defined by wet etching the W between the Au probing electrodes using H_2_O_2_ for 60 s, followed by NH_4_OH for 60 s both at a heated temperature of 60 °C. For the reference sample, RTP was performed at 700 °C for 30 s in a N_2_ environment, followed by UV-lithography and selective wet etching the W to define the capacitors.

### Electrical Characterization

Electrical characterization was performed in an MPI TS2000-SE probe station by using a Keysight B1500A parameter analyzer equipped with a B1530A waveform generator fast measurement unit (WGFMU) for pulsed measurements. For current–voltage measurements, high-resolution source measurement units (HRSMUs) coupled with E5288A Atto-sense units were used. The electrical characterization was performed on circular capacitors with a radius of 5 μm. The conventional positive-up–negative-down (PUND) technique was utilized to measure the polarization versus electric field (P–E) characteristics. Rectangular voltage pulses were used for wake-up cycling and endurance measurements at a frequency of 100 kHz. All PUND measurements were performed at 10 kHz.

### Material Characterization

Nanofocused X-ray diffraction (nano-XRD) measurements were performed at the NanoMAX beamline of the MAX IV Laboratory (Lund, Sweden). Diffraction measurements were performed at a 10° incidence using a 73 nm × 73 nm focused X-ray probe at 12 keV. Diffraction patterns were collected on a Pilatus3 1 M wide-angle scattering detector with a 172-μm pixel size at a distance of 1.49 m. The detector distance and scattering angles were calibrated using a lanthanum hexaboride (LaB_6_) polycrystalline reference sample.

X-ray photoelectron spectroscopy (XPS) was performed at the FlexPES beamline of the MAX IV Laboratory. XPS was performed for Ti 2p, N 1s, Zr 3d, Hf 4f, and O 1s core levels at varying photon energies, resulting in varying kinetic energies and probing depths. Spectral deconvolution and quantification enable a thorough evaluation of the changes in interface chemistry due to the annealing treatment. For this, singlets (for N 1s and O 1s) and doublets (for Ti 2p, Zr 3d, and Hf 4f) of the different components were fitted by Voigt functions after subtracting a Shirley background using the IgorPro software. Fitting parameters are listed in Table S1. The stated IMFP in Figure 4 was calculated using the works by Tanuma et al. and Powell et al.^48,49^

The thin-film topography and piezoresponse were measured by using a Bruker Dimension Icon atomic force microscope (AFM) in air ambiance. Piezoresponse force spectroscopy (PFS) measurements were performed with a conductive Pt/Ir-coated probe tip (SCM-PIT-V2, Bruker). All PFS measurements were done subresonance and using a *V*_ac_ of 500 mV. To help eliminate long-range electrostatic contributions, the electrostatic blind spot (ESBS) concept was exploited.^50^

## Data Availability

The datasets analyzed in this study are available from the corresponding authors upon reasonable request.

## Supporting Information Available

Details on the NLA temperature simulations; P–E of the device with the largest 2*P*_r_; measured electrical characteristics of a thermally annealed reference sample; explanation of the tungsten diffraction ring found in Figure 3; nano-XRD HZO peak fitting; piezoresponse force microscopy measurements; X-ray photoelectron spectroscopy fitting details (PDF)

## References

[ref1] Müller J.; Böscke T. S.; Schröder U.; Mueller S.; Bräuhaus D.; Böttger U.; Frey L.; Mikolajick T. Ferroelectricity in Simple Binary ZrO2 and HfO2. Nano Lett. 2012, 12 (8), 4318–4323. 10.1021/nl302049k. 22812909

[ref2] Shibayama S.; Nishimura T.; Migita S.; Toriumi A. Thermodynamic Control of Ferroelectric-Phase Formation in Hf_x_Zr_1– x_O_2_ and ZrO_2_. J. Appl. Phys. 2018, 124 (18), 184101 10.1063/1.5028181.

[ref3] Hyuk Park M.; Joon Kim H.; Jin Kim Y.; Lee W.; Moon T.; Seong Hwang C. Evolution of Phases and Ferroelectric Properties of Thin Hf_0.5_Zr_0.5_O_2_ Films According to the Thickness and Annealing Temperature. Appl. Phys. Lett. 2013, 102 (24), 242905 10.1063/1.4811483.

[ref4] Lehninger D.; Ali T.; Olivo R.; Lederer M.; Kampfe T.; Mertens K.; Seidel K. Furnace Annealed HfO 2 -Films for the Integration of Ferroelectric Functionalities into the BEoL. In 2020 Joint Conference of the IEEE International Frequency Control Symposium and International Symposium on Applications of Ferroelectrics (IFCS-ISAF); IEEE, 2020; pp 1 3.

[ref5] Mikolajick T.; Park M. H.; Begon-Lours L.; Slesazeck S. From Ferroelectric Material Optimization to Neuromorphic Devices. Adv. Mater. 2023, 35 (37), 2206042 10.1002/adma.202206042. 36017895

[ref6] Mikolajick T.; Schroeder U.; Lomenzo P. D.; Breyer E. T.; Mulaosmanovic H.; Hoffmann M.; Mittmann T. Next Generation Ferroelectric Memories Enabled by Hafnium Oxide. 2019 IEEE International Electron Devices Meeting (IEDM); IEEE, 2019, 354 357.

[ref7] Park J. Y.; Choe D.-H.; Lee D. H.; Yu G. T.; Yang K.; Kim S. H.; Park G. H.; Nam S.-G.; Lee H. J.; Jo S.; et al. Revival of Ferroelectric Memories Based on Emerging Fluorite-Structured Ferroelectrics. Adv. Mater. 2023, 35 (43), 2204904 10.1002/adma.202204904. 35952355

[ref8] Hwang J.; Goh Y.; Jeon S. Physics, Structures, and Applications of Fluorite-Structured Ferroelectric Tunnel Junctions. Small 2023, 20, 2305271 10.1002/smll.202305271. 37863823

[ref9] Ali T.; Olivo R.; Kerdiles S.; Lehninger D.; Lederer M.; Sourav D.; Royet A.-S.; Sunbul A.; Prabhu A.; Kuhnel K., Study of Nanosecond Laser Annealing on Silicon Doped Hafnium Oxide Film Crystallization and Capacitor Reliability. In 2022 IEEE International Memory Workshop (IMW); IEEE, 2022; pp 1 4.

[ref10] Song M. S.; Park K.; Lee K.; Cho J. W.; Lee T. Y.; Park J.; Chae S. C. Selective Crystallization of Ferroelectric Hf x Zr 1– x O 2 via Excimer Laser Annealing. ACS Appl. Electron. Mater. 2023, 5 (1), 117–122. 10.1021/acsaelm.2c01555.

[ref11] Grenouillet L.; Francois T.; Coignus J.; Kerdiles S.; Vaxelaire N.; Carabasse C.; Mehmood F.; Chevalliez S.; Pellissier C.; Triozon F., Nanosecond Laser Anneal (NLA) for Si-Implanted HfO2 Ferroelectric Memories Integrated in Back-End of Line (BEOL). In 2020 IEEE Symposium on VLSI Technology; IEEE, 2020; Vol. 2020, pp 1 2.

[ref12] Chen L.; Song W.; Wang W.; Lee H. K.; Chen Z.; Zhao W.; Zhu Y. KrF Excimer Laser Annealing With an Ultra-Low Laser Fluence for Enabling Ferroelectric HfZrO. IEEE Electron Device Lett. 2023, 44 (1), 32–35. 10.1109/LED.2022.3223109.

[ref13] Volodina N.; Dmitriyeva A.; Chouprik A.; Gatskevich E.; Zenkevich A. Ferroelectric Hf_0.5_Zr_0.5_O_2_ Thin Films Crystallized by Pulsed Laser Annealing. Phys. Status Solidi RRL 2021, 15 (5), 2100082 10.1002/pssr.202100082.

[ref14] Tabata T.; Halty S.; Rozé F.; Huet K.; Mazzamuto F. Non-Doped HfO 2 Crystallization Controlled by Dwell Time in Laser Annealing. Appl. Phys. Express 2021, 14 (11), 115503 10.35848/1882-0786/ac2c18.

[ref15] Frechilla A.; Napari M.; Strkalj N.; Barriuso E.; Niang K.; Hellenbrand M.; Strichovanec P.; Simanjuntak F. M.; Antorrena G.; Flewitt A.; et al. Spatially Selective Crystallization of Ferroelectric Hf0.5Zr0.5O2 Films Induced by Sub-Nanosecond Laser Annealing. Appl. Mater. Today 2024, 36, 102033 10.1016/j.apmt.2023.102033.

[ref16] Tian X.; Shibayama S.; Nishimura T.; Yajima T.; Migita S.; Toriumi A. Evolution of Ferroelectric HfO_2_ in Ultrathin Region down to 3 Nm. Appl. Phys. Lett. 2018, 112 (10), 102902 10.1063/1.5017094.

[ref17] Schroeder U.; Park M. H.; Mikolajick T.; Hwang C. S. The Fundamentals and Applications of Ferroelectric HfO2. Nat. Rev. Mater. 2022, 7 (8), 653–669. 10.1038/s41578-022-00431-2.

[ref18] Park M. H.; Shimizu T.; Funakubo H.; Schroeder U. Structural Origin of Temperature-Dependent Ferroelectricity. In Ferroelectricity in Doped Hafnium Oxide: Materials, Properties and Devices; Elsevier Ltd., 2019; pp. 193 216.

[ref19] Park M. H.; Lee Y. H.; Hwang C. S. Understanding Ferroelectric Phase Formation in Doped HfO 2 Thin Films Based on Classical Nucleation Theory. Nanoscale 2019, 11 (41), 19477–19487. 10.1039/C9NR05768D. 31549704

[ref20] Jo S.; Lee H.; Choe D.-H.; Kim J.-H.; Lee Y. S.; Kwon O.; Nam S.; Park Y.; Kim K.; Chae B. G.; Kim S.; Kang S.; Moon T.; Bae H.; Won J. Y.; Yun D.-J.; Jeong M.; Lee H. H.; Cho Y.; Lee K.-H.; Lee H. J.; Lee S.; Nam K.-J.; Jung D.; Kuh B. J.; Ha D.; Kim Y.; Park S.; Kim Y.; Lee E.; Heo J. Negative Differential Capacitance in Ultrathin Ferroelectric Hafnia. Nat. Electron. 2023, 6 (5), 390–397. 10.1038/s41928-023-00959-3.

[ref21] Kim S. J.; Mohan J.; Lee J.; Lee J. S.; Lucero A. T.; Young C. D.; Colombo L.; Summerfelt S. R.; San T.; Kim J. Effect of film thickness on the ferroelectric and dielectric properties of low-temperature (400 °C) Hf0.5Zr0.5O2 films. Appl. Phys. Lett. 2018, 112 (17), 172902 10.1063/1.5026715.

[ref22] Böscke T. S.; Müller J.; Bräuhaus D.; Schröder U.; Böttger U. Ferroelectricity in Hafnium Oxide Thin Films. Appl. Phys. Lett. 2011, 99 (10), 102903 10.1063/1.3634052.

[ref23] Böscke T. S. Crystalline Hafnia and Zirconia Based Dielectrics for Memory Applications Cuvillier Verla, 2010.

[ref24] Athle R., Ferroelectric Memristors - Materials, Interfaces and Applications, Doctoral Thesis, Lund University 2024.

[ref25] Datta S.; Dutta S.; Grisafe B.; Smith J.; Srinivasa S.; Ye H. Back-End-of-Line Compatible Transistors for Monolithic 3-D Integration. IEEE Micro 2019, 39 (6), 8–15. 10.1109/MM.2019.2942978.

[ref26] Böscke T. S. Crystalline Hafnia and Zirconia Based Dielectrics for Memory Applications. Doktor Dissertation, Vom Promotionsausschuss: Berlin-Charlottenburg 2018.

[ref27] Francois T.; Grenouillet L.; Coignus J.; Vaxelaire N.; Carabasse C.; Aussenac F.; Chevalliez S.; Slesazeck S.; Richter C.; Chiquet P.; et al. Impact of Area Scaling on the Ferroelectric Properties of Back-End of Line Compatible Hf_0.5_Zr_0.5_O_2_ and Si: HfO_2_-Based MFM Capacitors. Appl. Phys. Lett. 2021, 118 (6), 062904 10.1063/5.0035650.

[ref28] Francois T.; Pellissier C.; Slesazeck S.; Havel V.; Richter C.; Makosiej A.; Giraud B.; Breyer E. T.; Materano M.; Chiquet P., Demonstration of BEOL-Compatible Ferroelectric Hf 0.5 Zr 0.5 O 2 Scaled FeRAM Co-Integrated with 130nm CMOS for Embedded NVM Applications. In 2019 IEEE International Electron Devices Meeting (IEDM); IEEE, 2019; pp 15.7.1 15.7.4.

[ref29] Persson A. E. O.; Athle R.; Littow P.; Persson K.-M.; Svensson J.; Borg M.; Wernersson L.-E. Reduced Annealing Temperature for Ferroelectric HZO on InAs with Enhanced Polarization. Appl. Phys. Lett. 2020, 116 (6), 062902 10.1063/1.5141403.

[ref30] Athle R.; Persson A. E.; Troian A.; Borg M. Top Electrode Engineering for Freedom in Design and Implementation of Ferroelectric Tunnel Junctions Based on Hf 1– x Zr x O 2. ACS Appl. Electron. Mater. 2022, 4 (3), 1002–1009. 10.1021/acsaelm.1c01181.

[ref31] Goh Y.; Hwang J.; Lee Y.; Kim M.; Jeon S. Ultra-Thin Hf 0.5 Zr 0.5 O 2 Thin-Film-Based Ferroelectric Tunnel Junction via Stress Induced Crystallization. Appl. Phys. Lett. 2020, 117 (24), 242901 10.1063/5.0029516.

[ref32] Cheema S. S.; Kwon D.; Shanker N.; dos Reis R.; Hsu S. L.; Xiao J.; Zhang H.; Wagner R.; Datar A.; McCarter M. R.; Serrao C. R.; Yadav A. K.; Karbasian G.; Hsu C. H.; Tan A. J.; Wang L. C.; Thakare V.; Zhang X.; Mehta A.; Karapetrova E.; Chopdekar R. V.; Shafer P.; Arenholz E.; Hu C.; Proksch R.; Ramesh R.; Ciston J.; Salahuddin S. Enhanced Ferroelectricity in Ultrathin Films Grown Directly on Silicon. Nature 2020, 580 (7804), 478–482. 10.1038/s41586-020-2208-x. 32322080

[ref33] Grimley E. D.; Schenk T.; Sang X.; Pešić M.; Schroeder U.; Mikolajick T.; LeBeau J. M. Structural Changes Underlying Field-Cycling Phenomena in Ferroelectric HfO 2 Thin Films. Adv. Electron. Mater. 2016, 2 (9), 1600173 10.1002/aelm.201600173.

[ref34] Yurchuk E.; Muller J.; Muller S.; Paul J.; Pesic M.; van Bentum R.; Schroeder U.; Mikolajick T. Charge-Trapping Phenomena in HfO 2 -Based FeFET-Type Nonvolatile Memories. IEEE Trans. Electron Devices 2016, 63 (9), 3501–3507. 10.1109/TED.2016.2588439.

[ref35] Hamouda W.; Pancotti A.; Lubin C.; Tortech L.; Richter C.; Mikolajick T.; Schroeder U.; Barrett N. Physical Chemistry of the TiN/Hf 0.5 Zr 0.5 O 2 Interface. J. Appl. Phys. 2020, 127 (6), 064105 10.1063/1.5128502.

[ref36] Athle R.; Blom T.; Irish A.; Persson A. E. O.; Wernersson L.; Timm R.; Borg M. Improved Endurance of Ferroelectric Hf x Zr 1–x O 2 Integrated on InAs Using Millisecond Annealing. Adv. Mater. Interfaces 2022, 9 (12), 2201038 10.1002/admi.202201038.

[ref37] Park M. H.; Lee Y. H.; Mikolajick T.; Schroeder U.; Hwang C. S. Review and Perspective on Ferroelectric HfO2-Based Thin Films for Memory Applications. MRS Commun. 2018, 8 (3), 795–808. 10.1557/mrc.2018.175.

[ref38] Lyu J.; Song T.; Fina I.; Sánchez F. High Polarization, Endurance and Retention in Sub-5 Nm Hf 0.5 Zr 0.5 O 2 Films. Nanoscale 2020, 12 (20), 11280–11287. 10.1039/D0NR02204G. 32420576

[ref39] O’Connor É.; Halter M.; Eltes F.; Sousa M.; Kellock A.; Abel S.; Fompeyrine J. Stabilization of Ferroelectric HfxZr1–xO2 Films Using a Millisecond Flash Lamp Annealing Technique. APL Mater. 2018, 6 (12), 121103 10.1063/1.5060676.

[ref40] Chouprik A.; Zakharchenko S.; Spiridonov M.; Zarubin S.; Chernikova A.; Kirtaev R.; Buragohain P.; Gruverman A.; Zenkevich A.; Negrov D. Ferroelectricity in Hf 0.5 Zr 0.5 O 2 Thin Films: A Microscopic Study of the Polarization Switching Phenomenon and Field-Induced Phase Transformations. ACS Appl. Mater. Interfaces 2018, 10 (10), 8818–8826. 10.1021/acsami.7b17482. 29464951

[ref41] Kashir A.; Ghiasabadi Farahani M.; Kamba S.; Yadav M.; Hwang H. Hf 1– x Zr x O 2 /ZrO 2 Nanolaminate Thin Films as a High-κ Dielectric. ACS Appl. Electron. Mater. 2021, 3 (12), 5632–5640. 10.1021/acsaelm.1c01105.

[ref42] Rowtu S.; Meihar P.; Pandey A.; Ali M. H.; Lashkare S.; Ganguly U. Interlayer-Engineered Local Epitaxial Templating Induced Enhancement in Polarization (2 P *r* > 70 μ C/Cm 2) in Hf 0.5 Zr 0.5 O 2 Thin Films. IEEE Trans. Electron Devices 2023, 70 (7), 3536–3541. 10.1109/TED.2023.3277804.

[ref43] Filatova E. O.; Konashuk A. S.; Sakhonenkov S. S.; Sokolov A. A.; Afanas’ev V. V. Re-Distribution of Oxygen at the Interface between γ-Al2O3 and TiN. Sci. Rep. 2017, 7 (1), 4541 10.1038/s41598-017-04804-4. 28674397 PMC5495785

[ref44] Yong Z.; Persson K.-M.; Saketh Ram M.; D’Acunto G.; Liu Y.; Benter S.; Pan J.; Li Z.; Borg M.; Mikkelsen A.; et al. Tuning Oxygen Vacancies and Resistive Switching Properties in Ultra-Thin HfO2 RRAM via TiN Bottom Electrode and Interface Engineering. Appl. Surf. Sci. 2021, 551, 149386 10.1016/j.apsusc.2021.149386.

[ref45] Athle R.; Persson A. E. O.; Irish A.; Menon H.; Timm R.; Borg M. Effects of TiN Top Electrode Texturing on Ferroelectricity in Hf 1– x Zr x O 2. ACS Appl. Mater. Interfaces 2021, 13 (9), 11089–11095. 10.1021/acsami.1c01734. 33625827 PMC8027987

[ref46] Milošv I.; Strehblow H.-H.; Navinšek B.; Metikoš-Huković M. Electrochemical and Thermal Oxidation of TiN Coatings Studied by XPS. Surf. Interface Anal. 1995, 23 (7–8), 529–539. 10.1002/sia.740230713.

[ref47] Sales M. G.; Jaszewski S. T.; Fields S. S.; Litwin P. M.; Ihlefeld J. F.; McDonnell S. J. Thermal Stability of Hafnium Zirconium Oxide on Transition Metal Dichalcogenides. Appl. Surf. Sci. 2021, 546, 149058 10.1016/j.apsusc.2021.149058.

[ref48] Tanuma S.; Powell C. J.; Penn D. R. Calculation of Electron Inelastic Mean Free Paths (IMFPs) VII. Reliability of the TPP-2M IMFP Predictive Equation. Surf. Interface Anal. 2003, 35 (3), 268–275. 10.1002/sia.1526.

[ref49] Powell C. J.; Jablonski A. NIST Electron Inelastic-Mean-Free-Path Database, NIST, 2010.

[ref50] Killgore J. P.; Robins L.; Collins L. Electrostatically-Blind Quantitative Piezoresponse Force Microscopy Free of Distributed-Force Artifacts. Nanoscale Adv. 2022, 4 (8), 2036–2045. 10.1039/D2NA00046F. 36133417 PMC9418616

