# Supplementary material for: Ferroelectricity in Ultrathin HfO2-Based Films by Nanosecond Laser Annealing
Source: ACS Appl Mater Interfaces. 2024 Oct 3;16(41):55684–92. doi: 10.1021/acsami.4c10002 (PMC11492164; doi:10.1021/acsami.4c10002)
Supplement: Supplementary file 1 — am4c10002_si_001.pdf [file am4c10002_si_001.pdf]

Supporting Information

Ferroelectricity in Ultra-thin HfO_2-based Films by Nanosecond Laser Annealing

Robin Athle[1,5*], *Megan O Hill*[2,3,5], *Austin Irish*[2,5], *Huaiyu Chen*[2,5], *Rainer Timm*[2,5], *Elias Kristensson*[4,6], *Jesper Wallentin*[2,5], *and Mattias Borg*[1,5*]

[1] Electrical and Information Technology, Lund University, Box 118, 22 100 Lund, Sweden

[2] Division of Synchrotron Radiation Research, Lund University, Box 118, 22 100 Lund, Sweden

[3] MAX IV Laboratory, Lund University Box 118, 22 100 Lund, Sweden

[4]Division of Combustion Physics, Lund University, Box 118, 22 100 Lund, Sweden

[5] NanoLund, Lund University, Box 118, 22 100 Lund, Sweden

[6] Lund Laser Center, Lund University, Box 118, 22 100 Lund, Sweden

Corresponding Authors*: robin.atle@eit.lth.se, mattias.borg@eit.lth.se

NLA Temperature simulations

Finite element simulations of the thermal annealing of the material stack due to exposure of the pulsed laser were performed using COMSOL 6.1. A material stack identical to the experimental stack was defined with a manually defined two-dimensional square mesh, using rotational symmetry. In a first step the depth profile of light absorption was simulated, considering the laser wavelength (532 nm) and the tabulated dielectric functions of the materials. Material parameters for pure HfO_2 were used for the HZO film. We assume 50% reflectivity as typical for W at this wavelength.[1] From the simulations it is evident that the full laser energy (>99%) is absorbed within the 50 nm thick W top electrode layer, see Figure S1a. For simplicity we assume a spatially linearly varying heat source throughout the W layer in the second stage - the heat transport simulation.

As the temperature in the material stack is expected to vary across a large range of temperature, and we are interested in temperature profiles on a very small length scale (nm), we ensure to use temperature dependent parameters for the specific heat capacity (C_p) and thermal conductivity (κ) for the materials. These are shown for TiN and W in Figure S1c-d, as the values for these materials are the most relevant and vary most with temperature. The heat capacity (56 x 10^{-6} J/mol/K at 300 K) and thermal conductivity (0.75 W/m/K) values for HfO_2 are orders of magnitude smaller than for the metals and thus the precise values matter less for the results of the simulation.

The laser profile is simulated as a gaussian intensity profile $I(r) = \frac{2}{\pi\sigma^2}\exp\left(-\frac{2r^2}{\sigma^2}\right)$ with $\sigma = 21.5\ \mu m$, as well as being similarly gaussian in time, then with FWHM = 6 ns, i.e. $\sigma = 6\ [ns]/2.355$. The total energy deposited (1-3 µJ) is then dispersed across both the radial, temporal and (linear) depth profile of the heat source (Figure S1b), which acts as a heat source in the heat transport equation.

Simulations including SiO_2 spacer layer

The Si CMOS back-end-of-line (BEOL) consists of multiple layers of metal lines surrounded by low-density oxide/nitride dielectric layers. A BEOL-integrated ferroelectric capacitor would thus be separated from the underlying CMOS devices in the front-end by these layers. To estimate the thermal impact of the NLA process on the Si CMOS front end, we perform the same simulations as for our experimental samples but including a 100 nm SiO_2 layer in between the TiN layer and Si substrate. Figure S1e shows the temporal evolution of the temperature in the various layers of the stack, for a laser energy of 1.0 µJ. A general observation is that the low thermal conductivity of SiO2 (1.1 W/m/K) will lead to an additional thermal insulation, allowing the laser energy to be reduced by roughly 1.8x compared to our experimental samples while still reaching the same peak surface temperature. Secondly, the temperature of the capacitor layers (W, HZO and TiN) is very similar throughout the simulation, and importantly the cool-down rate of these layers is significantly longer compared to the experimental samples, reaching 500K only after roughly 100 ns. This is because cooling occurs mostly laterally in the metals, as the thermal resistance of the SiO_2 is much greater than in the metal layers. Finally, and most importantly from an integration perspective, the peak temperature in the top of the Si substrate never reaches higher than 430K. This means that the NLA process should not negatively affect the integrity of CMOS devices in the front-end. Figure S1f shows the depth profile at peak surface temperature for four different laser energies, clearly indicating that regardless of the surface temperature the Si remains at a very low temperature.

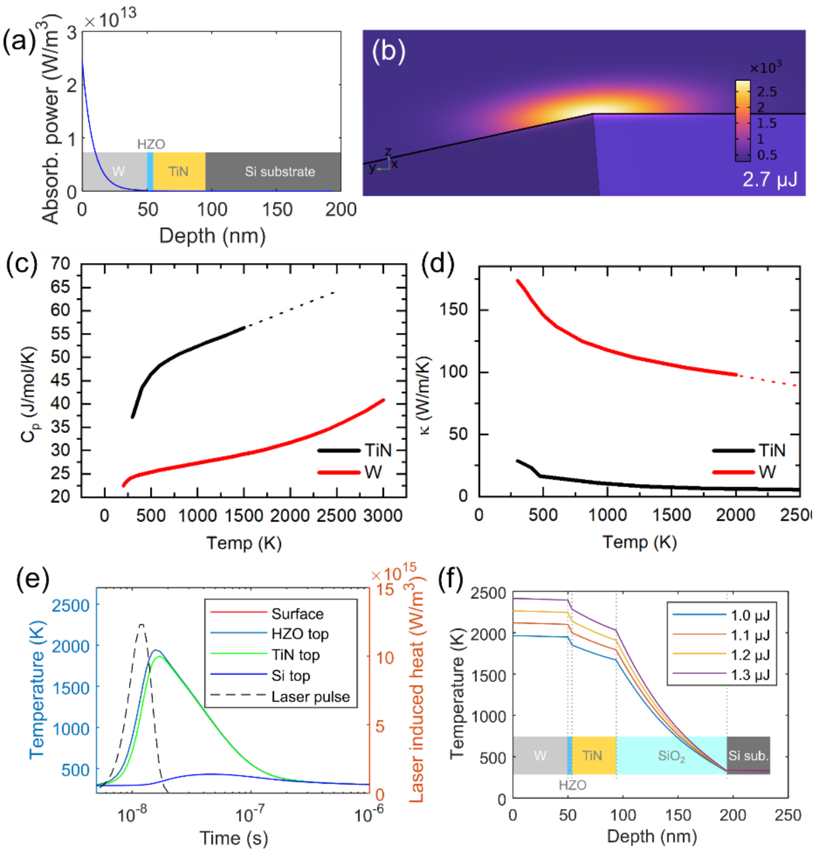

Figure S1. (a) Absorbed power as a function of depth into the material stack, highlighting that almost all of the laser power is absorbed in the W layer. (b) 3D plot of the gaussian temperature profile caused by the laser beam. The cut-out also highlights that the heating is extremely localized to the surface. Temperature dependent specific heat capacity (c) and thermal conductivity (d) used for the heat transport simulations. Dashed lines indicate linear extrapolation of the values to higher temperatures. (e-f) Simulation results including a 100 nm SiO_2 layer to isolate the ferroelectric capacitors from the Si front-end. (e) The evolution of the temperature at various depths using a laser pulse temperature of 1.0 μJ. (f) The depth profiles at peak temperature for various laser energies, indicating the strong thermal isolation provided by the SiO_2 layer.

Electrical characteristics of RTP reference sample

Figure S2 show the electrical characteristics of the RTP reference sample annealed at 700 °C for 30s in a N_2 environment. Figure S3a shows the polarization-electric field hysteresis curve where a P_r of 10 μC/cm^2 is achieved. Additionally, in Figure S3b the polarization switching endurance is presented. In contrast to the NLA device, a much more rapid wake-up process is observed owing to the longer time scale in RTP which allows for the grain growth. Despite a higher P_r, the film experiences hard breakdown after 10^6 switching cycles, one order of magnitude lower than NLA treated devices, indicating a higher defect density in the RTP annealed HZO film.

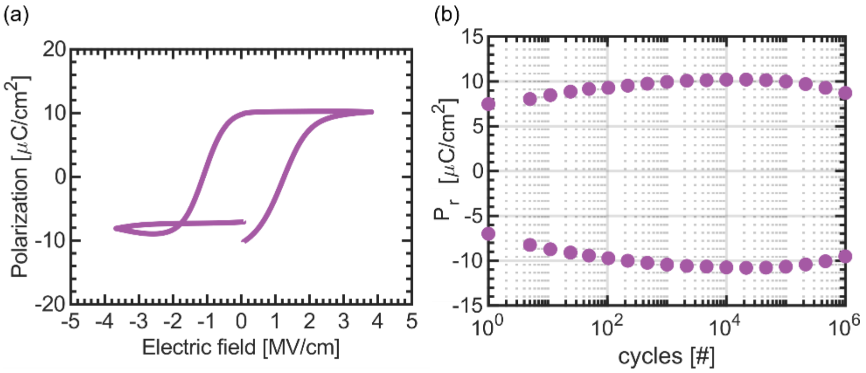

Figure S2. Polarization characteristics of RTP reference sample. (a) The PE hysteresis curve and (b) the endurance of the polarization switching.

P-E curve of the device with the largest $2P_r$

Figure S3 shows the P-E curves for the device with the largest $2P_r$ with cycling (1-10^7 cycles).

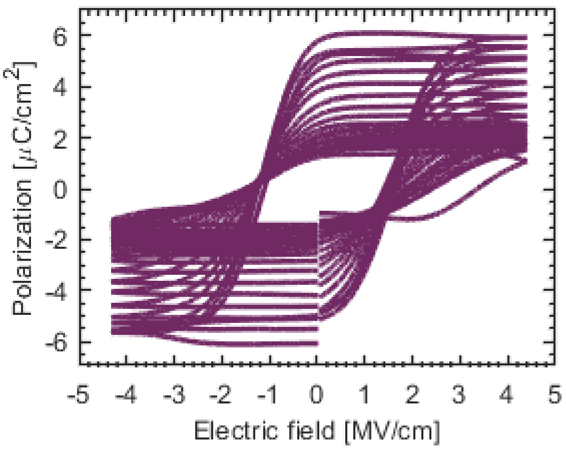

Figure S3. Evolution of the P-E curve with cycling for the device with the largest $2P_r$ achieved by NLA.

Tungsten Diffraction in XRD

Figure 3(d, f, g) show a diffracting ring around the device contact associated with W (110) scattering. The presence of W outside of the device contact is unexpected. However, since the signal forms a

ring around the contact in the approximate size of the laser annealing spot, we can hypothesize that this additional W diffraction results from a diffusion process during laser annealing. During the laser annealing process, the W top electrode can reach temperatures up to 2500 K, which is ~0.65 Tm of Tungsten. Such high temperature may allow for W surface diffusion within the laser spot, which is locked in place on the lower-temperature exterior of the X-ray profile. Alternatively, and more likely, this is an effect of Au diffusion (Tm= 1,337 K). Though the laser profile is only 50 µm FHWM, the tails of the laser spot may be high enough temperature to cause diffusion from the Au alignment markers (50 µm from the laser center). This diffused Au sitting at the edges of the laser profile prevent the etching of W, which is selectively etched following the annealing procedure. This can be prevented by spacing Au alignment markers further from the laser annealing spot or using an alignment metal with a higher diffusion temperature.

Nano-XRD HZO Peak Fitting

The ROI (within the white dashed line) in Figure 3f indicates the region where HZO has crystallized. Summing the diffraction patterns over this area and integrating across the azimuthal angle (χ) reveals a strong HZO associated peak around 20°. This peak overlaps with o-phase (111) expected for HZO and t-phase (011) expected for HfO_2. Attempting to deconvolve these contributions, the peak was fit to two pseudo-Voigt functions using non-linear least-squares curve fitting. Given the potential error in the peak positions and FWHM, the variation in fitting parameters is too large and the peak overlap is too significant to determine a percentage contribution from each crystal phase.

However, some approximation about the overall contribution can be made. Tetragonal peak positions were taken from the calculated positions of t-phase HfO_2 from The Materials Project [2] (20.29° at 12 keV) and calculated o-phase HZO from Ref. [3] (20.054° at 12 keV). Figure S4a shows the best fit fixed peak positions, giving 64% o-phase and 36% t-phase. The best fit, if the peak positions are allowed to fluctuate by 5%, is shown in Figure S4b, giving a lower phase content of t-phase (24%). Though we cannot confidently determine the overall phase contribution, no reasonable fitting produces a t-phase content over 40%. From this we can determine that the HZO peak is primarily composed of orthorhombic phase.

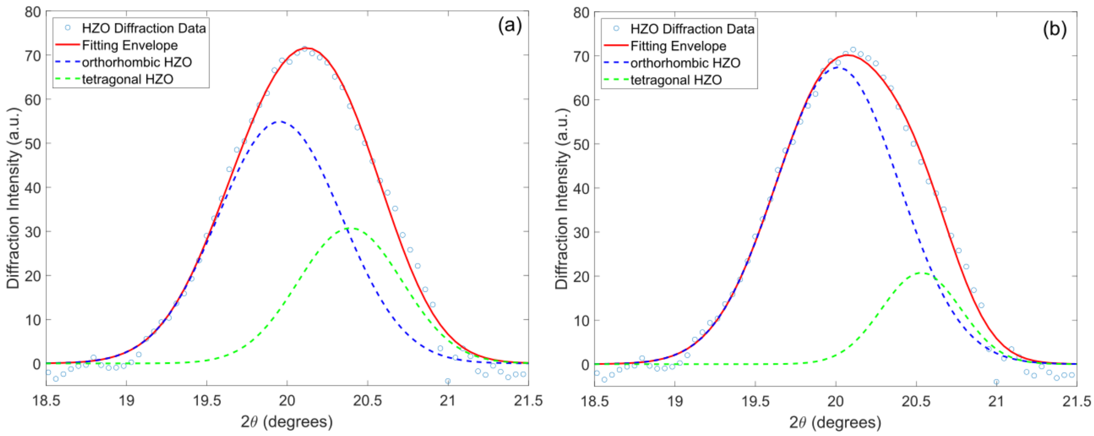

Figure S4. Least squares fitting of HZO diffraction peak from device C. (a) Fitting for fixed o-phase (blue) and t-phase (green) peak positions, (b) fitting for variable (up to 5%) peak positions.

Piezoresponse force spectroscopy

The piezoresponse force spectroscopy (PFS) measurements performed to verify the spatial selectivity of the NLA approach is presented in Figure S5. PFS was performed in two regions I and II, in close proximity to the electrodes of devices C and A. Figure S5a shows the topography data of an overview scan for device C with region I indicated by the white box. Figures S5b,d shows the phase and amplitude of the PFS done in region I, where HZO o(111) crystallization was confirmed by nano-XRD. The phase shift of 180° and expected coercive voltages of ±1 V are indicative of polarization switching in this area. In contrast, in region II no phase loop is observed (Figure S5c) and the measured amplitude (Figure S5e) is basically DC voltage independent, consistent with the nano-XRD data that the HZO film is amorphous in this region.

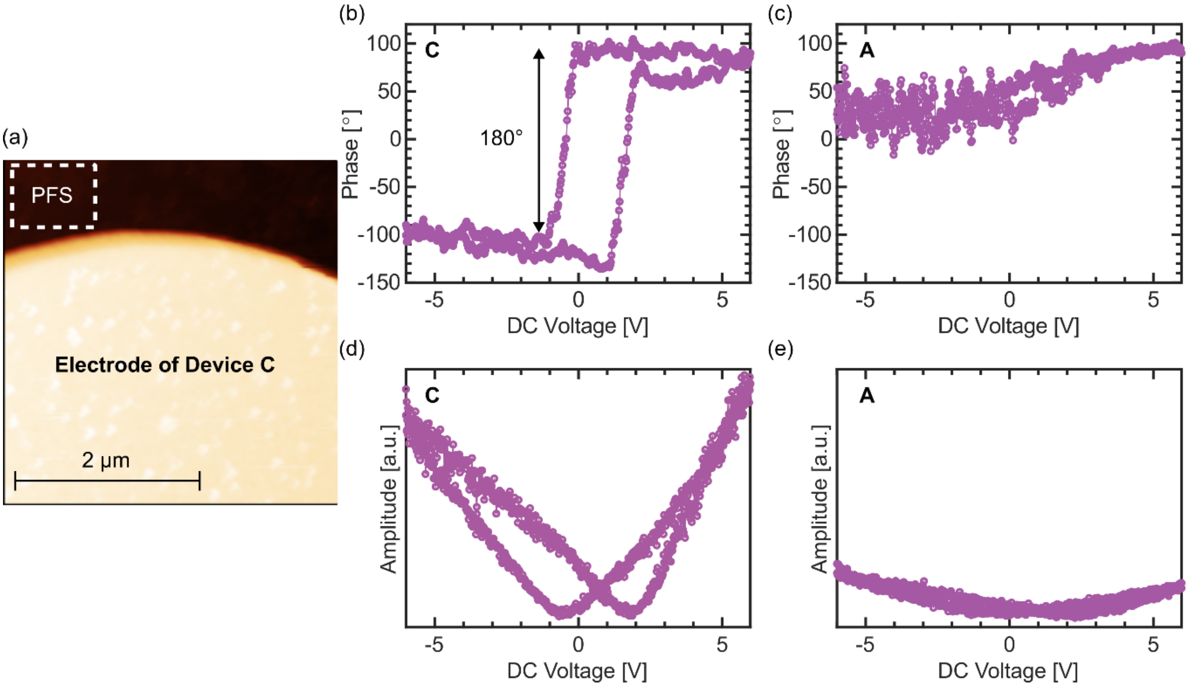

Figure S5. PFS measurements confirming the spatial selectivity of the NLA process. (a) Topography data from overview scan at device C, the white box highlights a region near the electrode where PFS measurements were done. (b)-(e) shows the measured phase (b,c) and amplitude (d,e) of the regions in close proximity to the C and A electrodes respectively.

XPS Fitting

Fitting of the N 1s core level spectra for the reference sample is shown in Figure S6. For other samples, the fitting parameters were constrained to be consistent with those from the reference as follows. Gaussian FWHM (± 0.1 eV) was fixed to 1.2 eV, 0.9 eV, 1.7 eV, and 1.7 eV and chemical shifts (± 0.1 eV) were fixed to 397.1 eV, 398.2 eV, 399.5 eV, and 401.1 eV for TiN, NO_1, NO_2, NO_3 respectively. Lorentzian FWHM were all fixed to 0.1 eV, TiN binding energy was aligned to 397.1 eV [4], and area was left free. Intensities were normalized to the X-ray beam, which was assumed to be proportional to the Hf 4f and O 1s signals which were in agreement. The results are shown below in Table S1. Results from Figure 4b in the main text were obtained by combining all area contributions from oxide components.

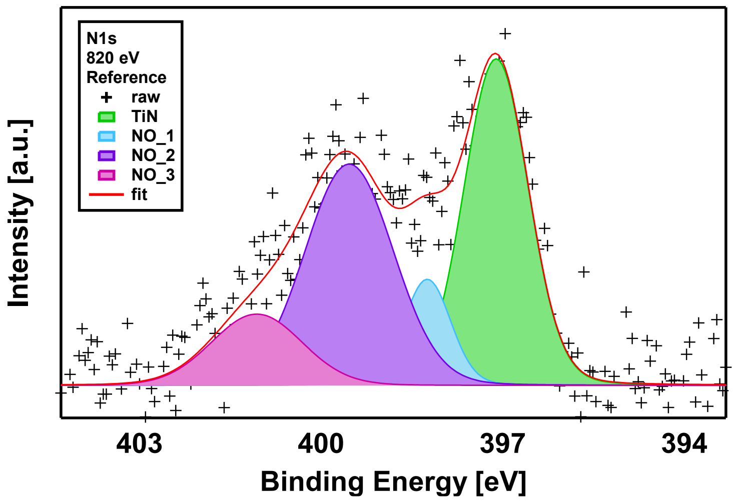

Figure S6. Example peak fitting of N 1s for the unannealed reference sample at 820 eV excitation.

Table S1 Fitting results from XPS of N 1s at 820 eV excitation.

	TiN			NO_1			NO_2			NO_3		
**Reference**	1.0	1.2	397.1	0.2	0.9	398.2	0.9	1.7	399.5	0.3	1.7	401.1
**1.2 µJ**	1.3	1.3	397.1	0.8	1	398.3	1.5	1.8	399.6	0.4	1.8	401.2
**1.8 µJ**	1.3	1.2	397.1	0.5	1	398.3	1.2	1.8	399.5	0.3	1.6	401.1
**2.4 µJ**	1.1	1.3	397.1	0.4	1	398.3	1.4	1.8	399.7	0.4	1.7	401.1
**1.2 µJ x4**	1.2	1.3	397.1	0.4	1	398.2	1.3	1.8	399.5	0.3	1.6	401.2
**1.2 µJ x16**	1.3	1.3	397.1	0.8	1	398.3	1.5	1.8	399.4	0.6	1.6	400.9
**1.2 µJ x64**	1.2	1.3	397.1	0.6	1	398.3	2.0	1.8	399.5	0.6	1.6	401
**RTP**	1.0	1.3	397.1	0.3	1	398.1	1.5	1.8	399.5	1.8	1.7	401
	**a.u.***	**eV ¤**	**eV ~**	***Area, ¤ Gaussian FWHM, ~ Binding Energy**								

References

1. G Vijaya et al 2016 IOP Conf. Ser.: Mater. Sci. Eng. 149 012075
2. Jain, Anubhav, et al. "Commentary: The Materials Project: A materials genome approach to accelerating materials innovation." *APL materials* 1.1 (2013).
3. Muller, Johannes, et al. "Ferroelectricity in simple binary ZrO2 and HfO2." *Nano letters* 12.8 (2012): 4318-4323.
4. Jaeger, Dominik, and Jörg Patscheider. "A complete and self-consistent evaluation of XPS spectra of TiN." *Journal of Electron Spectroscopy and Related Phenomena* 185.11 (2012): 523-534.
